# Long-term performance of three mesophilic anaerobic digesters to convert animal and agro-industrial wastes into organic fertilizer

**DOI:** 10.1016/j.jclepro.2021.127271

**Published:** 2021-07-20

**Authors:** Xiaoqian Zhang, Igor M. Lopes, Ji-Qin Ni, Yongping Yuan, Chi-Hua Huang, Douglas R. Smith, Indrajeet Chaubey, Shubiao Wu

**Affiliations:** a Department of Agricultural and Biological Engineering, Purdue University, West Lafayette, IN 47907, USA; b Key Laboratory of Clean Utilization Technology for Renewable Energy in Ministry of Agriculture, College of Engineering, China Agricultural University, Beijing, 100083, PR China; c USEPA-Office of Research and Development, Research Triangle Park, NC 27711, USA; d USDA-ARS, National Soil Erosion Research Lab, West Lafayette, IN 47907, USA; e Department of Agroecology, Aarhus University, Blichers Allé 20, 8830 Tjele, Denmark

**Keywords:** Anaerobic digestion, Ammonium nitrogen, Effluent, Nutrients, Metals, Total solids

## Abstract

Few studies have investigated the performance of anaerobic digestion (AD) to convert animal and agro-industrial wastes to organic fertilizers over a long-term field conditions. This paper studied three large-scale mesophilic digesters (D1eD3) over two years for their effects on feedstocks, which were dairy manure for D1 and D2 and co-digestion mixed manure and agro-industrial wastes for D3. Hydraulic retention times (HRT) were 9 d for D1, 12 d for D2, and 34 d for D3. Digester influent and effluent samples were taken every two months from the digesters and analyzed for pH, and concentrations of total solids (TS), ammonium nitrogen (NH_4_-N), total Kjeldahl nitrogen (TKN), total phosphorus (TP), and eight metals. The study revealed high variability in converting feedstock in the three digesters. Compared with their respective influent, the mean digester effluent pH decreased from 7.9 by 0.6 in D1 (*p* < 0.01) and by 0.3 in D2 (*p* < 0.01), but it increased from 6.1 by 1.8 in D3 (*p* < 0.01). The mean digester effluent TS increased from 3.4% by 0.1% (*p* > 0.05) in D1, but it decreased from 4.9% by 1.3% in D2 (*p* < 0.05) and from 12.3% by 4.8% in D3 (*p* < 0.01). All three digesters significantly increased NH_4_-N concentrations by 21.4 e81.8% (*p* < 0.05), but insignificantly changed TKN and TP concentrations (*p* > 0.05). Effects of AD on all metal concentrations were mixed and were insignificant (*p* > 0.05) because of large concentration variations. However, study of a ratio quotient (*q*_*Mg*_) using magnesium (Mg) as the reference discovered accumulation of NH_4_-N, copper, potassium, and sodium, but loss of TKN, TP, iron, manganese, zinc, and calcium during AD for D2 and D3. The impact of AD conversion was closely related with types of feedstock (on pH) and HRT (on TS and NH_4_-N). The results of this study can assist in developing strategies for cleaner production using AD in an environmentally sustainable manner.

## 1. Introduction

Anaerobic digestion (AD) has been increasingly adopted in the recent decades in the U.S. to produce biogas, a renewable energy, from agricultural and agro-industrial wastes (USEPA, 2020). Anaerobic digestion is carried out by a consortium of microorganisms that converts biodegradable organic matter into biogas. It induces biochemical changes to digester influent and generates a biologically more stable and partially hygienic effluent or digestate (Möller and Müller, 2012). Digester effluent usually contains a higher ratio of ammonium nitrogen (NH_4_-N) to total nitrogen (TN) and less decomposable organic matter, compared with the digester influent. It is also rich in phosphorus (P) and a variety of metals. Digester effluent can be a substitute for inorganic fertilizers and is a good soil amendment at croplands (e.g., Zhang et al., 2017). Agro-industrial wastes that usually cannot be directly applied to cropland, such as food processing wastes and biodiesel production byproducts (Page et al., 2015), can be converted into organic fertilizer through AD.

However, some studies have reported negative effects of effluent application on the environment. The NH_4_-N in the land-applied digestate can be transformed into nitrate (${\text{NO}}_{3}^{-}$) in the soil. This form of N can easily leach through the soil profile, causing pollution to groundwater. Wasteful N management can result in costs for human health and ecosystems (Van Grinsven et al., 2013). Additionally, cropland applied with digester effluent can lead to higher ammonia (NH_3_) emissions (Quakernack et al., 2012). While applying to the field, the infiltration of effluent into the soil can increase because of reduced total solids (TS) and viscosity, then the exchange surface of effluent with atmosphere can decrease. Even though, the increased NH_4_-N concentration and pH of the effluent may promote NH_3_ emissions during storage and field application (Möller, 2015). Excessive NH_3_ emissions to the atmosphere may cause eutrophication of N-deficient ecosystems and form ${\text{NH}}_{4}^{+}$ -based particulate matter in the air (Kai et al., 2008). Moreover, metals concentrated in the effluent due to the mass reduction during AD may generate an environmental risk by accumulating in the topsoil or in the food chain (Martin, 2008; Zhu et al., 2014). Consequently, effluent application in agriculture might also be restricted by metal contents in the effluent, such as copper (Cu) and zinc (Zn) (Alburquerque et al., 2012).

To better utilize the fertilizing values of digester effluent and protect soil, air, and water environment, it is essential to obtain sufficient understanding of the characteristics of the effluent, particularly under different field operating conditions and feedstocks. However, only limited information in this research area is available. Some studies reported N and P in effluent, but metals are rarely assessed (e.g., O’Brien et al., 2019). Additionally, though there have been many investigations into anaerobic digestion of agricultural wastes, most of those were conducted in laboratory conditions (e.g., Alburquerque et al., 2012). Moreover, the relationship among different constituents (such as nutrients, metals, pH, and TS) in effluent under field conditions has not been sufficiently studied.

Although field studies are less controlled compared with laboratory studies, field results are closer to the reality. Furthermore, establishing a large-scale anaerobic digester system requires millions of US dollars of investment and the system would be expected to remain in service for many years; but long-term field studies (>1 year) of digester effluent are extremely scarce. Therefore, in-field and long-term investigations are needed to gain new insights into the performance of AD systems on the conversion of agricultural and agro-industrial wastes into organic fertilizer.

The objective of the paper was to assess the changes in pH, TS, nutrients, and metals in digester influent and effluent at three large-scale mesophilic anaerobic digesters in the U.S. based on a 2year field study. Insights gained from this study can be used for digester management and post-digestion effluent treatment and utilization.

## 2. Materials and methods

### 2.1. Anaerobic digesters

Three anaerobic digesters located in Midwest, USA, were selected for the study. The digesters represented technologies for bioenergy production from animal wastes and agro-industrial wastes in the region. All three digesters were operated under mesophilic fermentation conditions at 35‒38 °C (Table 1).

Digester 1 (D1) had a plug flow design. It used dairy manure as the only feedstock and had a hydraulic retention time (HRT) of about 9 days.

Digester 2 (D2) was a Mixed Plug-Flow™ design by DVO Inc. (Chilton, WI, USA). Dairy manure was also the only feedstock for D2, which was operated at a relatively longer HRT of approximately 12 days.

Digester 3 (D3) also had a Mixed Plug-Flow design. Unlike D1 and D2, the feedstock of D3 was mixed manure from non-dairy livestock, and co-digestion wastes from agro-industries. The compositions and origin of agro-industries in D3 were confidential information for the industry and were not available for this paper. Digester D3 had a relatively longer HRT of about 34 days.

### 2.2. Sampling and sample analysis

Sampling of digester influent and effluent at the three digesters started in March 2013 and continued approximately every two months. After a few trials and methodology improvements, valid samples were obtained from July 2013 to May 2015. However, due to the digester maintenance, samples at D1 were not available for the first (January) and second (March) sampling events in 2014.

Composite samples were taken in the influent and effluent tanks at all three digesters. Each composite sample was composed of six subsamples that were taken at two different locations and three different depths in the digester influent and effluent pits: (1) within 0.3 m below the liquid surface, (2) at the midway of the liquid depth, and (3) about 0.3 m above the liquid bottom layer. Immediately after it was taken using a custom-designed slurry sampler, the subsample was placed in a plastic bucket. The sample pH and temperature (T) were measured using a MW102 portable pH meter (Milwaukee Instruments, Inc., Rocky Mount, NC, USA), which was calibrated before each sampling session. The subsamples were then mixed to get the composite sample in a cleaned bucket.

The composite sample was filled into three 500-mL polyethylene sample bottles while the liquid in the bucket being continuously mixed to ensure sample homogeneity. The sample in the first bottle was stored in its original state for total solids analysis. Sulfuric acid (H_2_SO_4_) was added in the second bottle to preserve the sample at pH < 2 for nutrient analysis. In the third bottle, nitric acid (HNO_3_) was added to preserve the sample at pH < 2 for metal analysis. The sample bottles were labeled and packaged with ice in a cooler to ensure that the sample T was below 4 °C, and shipped overnight to the U.S. Environmental Protection Agency (EPA) Chicago Regional Laboratory (CRL) for analysis.

The digester influent and effluent samples were analyzed in the CRL following the U.S. EPA methods (USEPA, 1983) for total solids (TS: EPA 160.3), total Kjeldahl nitrogen (TKN: EPA 351.2), ammonium nitrogen (NH_4_-N: EPA 350.1), total phosphorus (TP: EPA 365.4), and metals (EPA 200.7). Analyzed metals included potassium (K), calcium (Ca), sodium (Na), iron (Fe), magnesium (Mg), manganese (Mn), copper (Cu), and zinc (Zn).

### 2.3. Data processing and analysis

#### 2.3.1. Concentration and concentration relative to TS

The pH from on-site measurement and the CRL-reported concentrations (referred to as “concentrations”) of TS, nutrients and metals were used in the study. Differences in pH and concentrations among the three digesters in influent and effluent were investigated for variations in different digesters. The differences between influent and effluent of the same digester were studied for the conversion of feedstocks to effluent in the digesters. The characteristics of effluent from the three digesters were also compared with the reported effluent nutrient and metal concentrations in the literature.

Additionally, the concentrations were converted to concentrations related to TS (CRTS) based on the method used by Zirkler et al. (2014) to compare the variations in nutrients and metals among the three digesters. Assuming the specific weight of influent and effluent to be 1.0 kg L^−1^, the original unit of concentrations in mg L^−1^ were converted to mass fractions in mg kg^−1^. The CRTS were calculated with Eq. (1).

(1) $$CRTS=C_{m}/(10\cdot TS)$$

where *CRTS* is concentration related to TS, mg g^−1^; C_*m*_ is mass fraction of nutrient or metal converted from concentration of a sample, mg kg^−1^; *TS* is total solids of the sample, %.

Statistical *t*-test was used to compare the relevant data of pH, concentrations, and CRTS. The levels of significance were α = 0.01 and α = 0.05.

#### 2.3.2. Ratio quotient

A ratio quotient was used to analyze the nutrient and metal changes by comparing their concentrations of effluent with the respective influent for D2 and D3. Magnesium was chosen as the reference based on the study of Möller and Müller (2012) because Mg could keep constant during the AD. A quotient *q*_*Mg*_ of the influent ratio and the effluent ratio was calculated in this study as the index of accumulation and loss using Eq. (2), which was employed by Zirkler et al. (2014).

(2) $$q_{Mg}=\sum_{i=1}^{n}\frac{C_{Mg,IN_{i}}}{C_{NM,IN_{i}}}/\sum_{i=1}^{n}\frac{C_{Mg,EF_{i}}}{C_{NM,EF_{i}}}$$

where *q*_*Mg*_ is index of accumulation and loss, dimensionless; *n* is number of samples, n; C is concentration, mg L^−1^; subscripts *Mg* is Mg, *NM* is specific nutrient or metal, *IN* is influent, and *EF* is effluent.

A calculated *q*_*Mg*_ close to 1 indicated that no accumulation or loss had occurred. If the *q*_*Mg*_ was ≥1 or ≤1, an accumulation or loss of nutrient or metal, respectively, could have occurred during the AD.

#### 2.3.3. Regression

As one of the most popular approaches to modelling the relationship between a scalar response and one or more explanatory variables, linear regressions were performed to study the relationships between the nutrient or metal concentrations, and pH and/or TS of the effluent. Simple- and multiple linear regression models were used. Coefficients of determination (R^2^) and significant F values were used to test how strong the correlations among the parameters and the concentrations are. A linear regression equation was used for the analysis [Eq. (3)].

(3) $$C_{R}=A\cdot pH+B\cdot TS+C$$

where *C*_*R*_ is calculated sample concentration of specific nutrient or metal in regression, mg L^−1^; *A* and *B* are regression coefficients; *C* is regression constant; pH is mean pH of subsamples measured onsite, dimensionless; and *TS* is sample total solids, %. The coefficient *A* was assigned zero for simple linear regressions.

## 3. Results and discussion

### 3.1. Digester influent characteristics

#### 3.1.1. Influent pH and TS

The influent pH in the three digesters demonstrated a high variability (Table 2). The pH in D1 and D2 were both at 7.9 ± 0.3 (mean ± standard deviation), whereas the pH of 6.1 ± 0.4 in D3 was significantly lower (*p* < 0.05).

The TS concentrations of the influent to the three digesters were significantly different (*p* < 0.05) from each other. The D3 influent TS (12.3 ± 3.2%) was much greater than the influent to D1 and D2.

The different influent TS between D1 and D2 was most likely affected by manure handling methods (Sadaka and Engler, 2003), such as different mixing ratios of dairy manure with wastewater from milk parlors and dairy barn cleaning. The lower pH and higher TS in D3 could be related to the co-digestion using different feedstock, which included mixed animal manure and various agro-industrial wastes.

#### 3.1.2. Influent nutrients

The concentrations of nutrients (N and P) in influent varied among the three digesters, of which D1 had the lowest NH_4_-N, TKN, and TP values and D3 had the highest (Table 3). The differences were all statistically significant (*p* < 0.05). However, because D1 influent had the lowest TS, the nutrient CRTS in the three digesters did not differ significantly except for the TP. The influent to D3 had significantly higher TP than the other two digesters (*p* < 0.05). Because D1 and D2 both only used dairy manure from farms of similar production practice, conversion of concentrations to CRTS in this study provided an alternative approach to compare the nutrients in the feedstock.

#### 3.1.3. Influent metals

The eight metals (Cu, Fe, Mg, Mn, K, Na, Zn, and Ca) studied in this project also differed among the influent to the three digesters (Table 3). Metal concentrations that were different among the three digesters were Fe, K, and Ca (*p* < 0.05). The concentration differences of other five metals were more random among the three digesters.

However, comparison with CRTS provided a different picture. The CRTS between D1 and D2 influent were not statistically significant (*p* > 0.05) for six of the eight metals, except for Cu and Na, because both digesters used the same type of feedstock. Compared with D1 and D2, the CRTS of metals were all significantly different (*p* < 0.05) in D3 influent, except for K, of which the CRTS ranged from 35.5 ± 7.2 to 30.9 ± 17.6 mg g^−1^ for the three digesters.

Variations in the influent metal concentrations among the three digesters could also be related to different feedstocks. Most metals in manure were derived directly from the animal feed (Bolan et al., 2004). Several feed additives that contain metals were usually used to reduce the risk of disease outbreaks. For example, Cu was added as a footbath in milking yards to treat lameness in dairy cattle (Poulsen, 1998). Because most of the feed additives contain metals, it was likely to result in elevated concentrations of metals in manure (Nahm, 2002). Metal concentrations in manure could also depend on animal species (Miller et al., 1991).

### 3.2. Digester effluent characteristics

#### 3.2.1. Effluent pH and TS

Effluent samples from all three digesters had slightly alkaline pH, ranging from 7.3 ± 0.5 to 7.9 ± 0.4 (Table 2). There were significant variations in effluent pH between D1 and D3 (*p* < 0.05), and between D2 and D3 (*p* < 0.05), but not between D1 and D2 (*p* > 0.05). However, the effluent pH in all three digesters were close to the normal ranges of 7.8 ± 0.6 at digester systems fed with different feedstocks, ranging from chicken litter, cattle manure, pig manure to co-digestion of mixed manure, or straw plus manure, reported in the literature (Table 4).

Effluent TS for the three digesters ranged from 3.5 ± 1.0 to 7.5 ± 1.7% (Table 2). Although there was no significant difference between D1 and D2 effluent TS (*p* > 0.05), the D3 effluent TS was significantly greater than those from D1 and D2 (*p* < 0.05) (Table 2).

#### 3.2.2. Effluent nutrients

Effluent among the three digesters showed variations in nutrients as well (Table 5). The mean TKN and TP concentrations in effluent were not different (*p* > 0.05) between D1 and D2. These concentrations fell within the data range reported in the literature (Table 4). However, the D3 effluent concentrations of NH_4_-N (3863 ± 888 mg L^−1^), TKN (7440 ± 1434 mg L^−1^), and TP (2500 ± 510 mg L^−1^) (Table 5) all exceeded the corresponding values in the literature (Table 4) and were statistically different from D1 and D2 (*p* < 0.05).

Nevertheless, effluent NH_4_-N in CRTS was significantly different among the three digesters (*p* < 0.05). Similar to the influent characteristics, there was no significant difference in the CRTS of TKN and TP between effluent from D1 and D2 (*p* > 0.05). Consequently, the CRTS of D3 nutrients were significantly different from D1 and D2 (*p* < 0.05). This could also be attributed to the nutrient-rich co-digestion influent to D3.

Another characteristic of effluent nutrients was the relatively lower ratios of NH_4_-N: TKN concentrations that were 46%, 54%, and 52% for D1, D2, and D3, respectively (Table 5). The mean effluent NH_4_-N: TKN ratio in the reviewed 14 reports was 63.2% (Table 4). This could probably be related to the different digester designs and operational processes, such as HRT.

#### 3.2.3. Effluent metals

Effluent metal concentration variability among the three digesters was also obvious (Table 5). Higher concentrations of Cu were found in the effluent from D1 (17.6 ± 7.0 mg L^−1^) and D2 (13.5 ± 2.4 mg L^−1^) than from D3 (4.8 ± 1.3 mg L^−1^). Other metals that had higher concentrations in D1 and D2 than in D3 included Mg and Mn. The effluent metal concentrations that were lower in D1 and D2 than in D3 were Fe, K, Na, Zn, and Ca. Much lower Fe concentrations, relative to the other metals, were found in D1 (52.4 ± 12.1 mg L^−1^) and D2 (51.5 ± 12.8 mg L^−1^) compared with D3 (1270 ± 286 mg L^−1^). Concentrations of five out of the eight metals, except for Mg, Mn, and Zn, were not statistically different between D1 and D2 (*p* > 0.05). Two of the metals, Mg and Mn, were statistically different among all three digesters (*p* < 0.05). Generally, D3 effluent metal concentrations showed more differences from D1 and D2. A high variety of metal concentrations was also found in effluent from different feedstocks in the literature (Table 4).

Comparison with metal effluent CRTS showed smaller differences. No significant differences were found between D1 and D2 (*p* > 0.05) except for Zn (Table 5). Nevertheless, there were significant differences between D3 and the other two digesters (*p* < 0.05) except for K (with both digesters) and Zn (with D2).

The differences in effluent metal concentrations between D3 and the other two digesters could be due to different feedstocks to the three digesters. For example, higher Cu concentrations were found in D1 and D2 than in D3 influent (Table 3) because Cu was frequently used as additives to prevent dairy diseases and to stimulate livestock growth (Alburquerque et al., 2012). Similar concentration variabilities in other metals were also found in digester influent. Moreover, data with high standard deviations during the 2-year study demonstrated temporal variations in effluent metal concentrations of the digesters in this study. This result agreed with the study of Zirkler et al. (2014).

Metals from anaerobic digesters have a potential impact on plant production and soil health. When digester effluent was applied to cropland as an organic fertilizer, most of the Cu and Zn could be highly utilized, as they were essential for plant and microbial growth (Makádi et al., 2012). However, other metals at excessive concentrations could pose a risk to the environment and need to be carefully monitored (Kataki et al., 2017).

### 3.3. Changes from digester influent to effluent in AD

#### 3.3.1. Changes in pH

Compared with the influent pH, the effluent pH significantly (*p* < 0.01) decreased by 0.6 in D1 and by 0.3 in D2, but significantly increased (*p* < 0.01) by 1.8 in D3 (Table 2). The pH is one of the most important characteristics in effluent as organic fertilizer. The pH can considerably affect NH_3_ emission and nitrogen (N) loss from the effluent. This is because per unit increase in pH can increase NH_3_ concentration in aqueous phase approximately 10 fold up to pH 9 (Vlek and Stumpe, 1978). Aqueous phase NH_3_ concentration can be directly proportional to NH_3_ emission rate (Ni, 1999). Ammonia emission can occur during post-digestion effluent storage in lagoons, which can last for several months. It can also happen during effluent cropland application, which occurred mainly within the first 10 h (Quakernack et al., 2012). Compared with influent pH, D1 and D2 had reduced potentials of NH_3_ emission and N loss during post-digestion storage and land-application because the pH in D1 and D2 effluent decreased. However, D3 had increased the potentials because pH in D3 effluent increased.

A number of factors during AD could affect the change in pH in AD. As methane-forming bacteria in the digester consume the volatile acids and alkalinity is produced, the pH of the digestion liquid increases and then stabilizes (Gerardi, 2003). Digestion liquid pH could also be affected by the release of NH_3_ and carbon dioxide (CO_2_) from the liquid. Release of NH_3_ could decrease and release of CO_2_ could increase the liquid pH (Ni et al., 2000). The effects of different biochemical processes brought dynamic equilibrium in the liquid pH that was shown in the effluent samples. This study also demonstrated that the effluent pH for the three digesters was not primarily affected by the influent pH (Table 2).

#### 3.3.2. Changes in TS

Compared with the influent TS, the respective effluent TS did not have significant change for D1 (*p* > 0.05), but significantly decreased by 26.5% for D2 (*p* < 0.05) and 39.0% for D3 (*p* < 0.01) (Table 2). These differences might be related to the HRT of the three digesters (Teglia et al., 2011). An increase in HRT could boost solids removal and degradation in digesters (Rizvi et al., 2015). As expected, the TS reduction in D3 was the highest and this could be attributed to the longest HRT (34 days) of D3. Different effluent TS could influence post-digestion process, which often include solid-liquid separation, and utilization or final cropland application.

#### 3.3.3. Changes in nutrients

Anaerobic digestion also changed nutrient concentrations in effluent (Table 5) compared with respective influent (Table 3). Through AD, the NH_4_-N, TKN, and TP concentrations increased by 36.8% (*p* < 0.01),10.4%, and 5.1%, respectively, in D1. However, AD in D2 and D3 affected nutrients differently. Although NH_4_-N increased in both D2 (by 21.4%, *p* < 0.05) and D3 (by 81.8%, *p* < 0.01), TKN and TP both decreased in D2 (by −7.2% and −8.0%, respectively) and D3 (by −13.4% and −14.7%, respectively). A general trend in these changes appeared to be related to the digester HRT in the three digesters. The longest HRT in D3 was followed by the greatest increase in NH_4_-N and reduction in TKN and TP concentrations, although the changes in TKN and TP were not statistically significant (*p* > 0.05) for all three digesters.

**Fig. 1.**
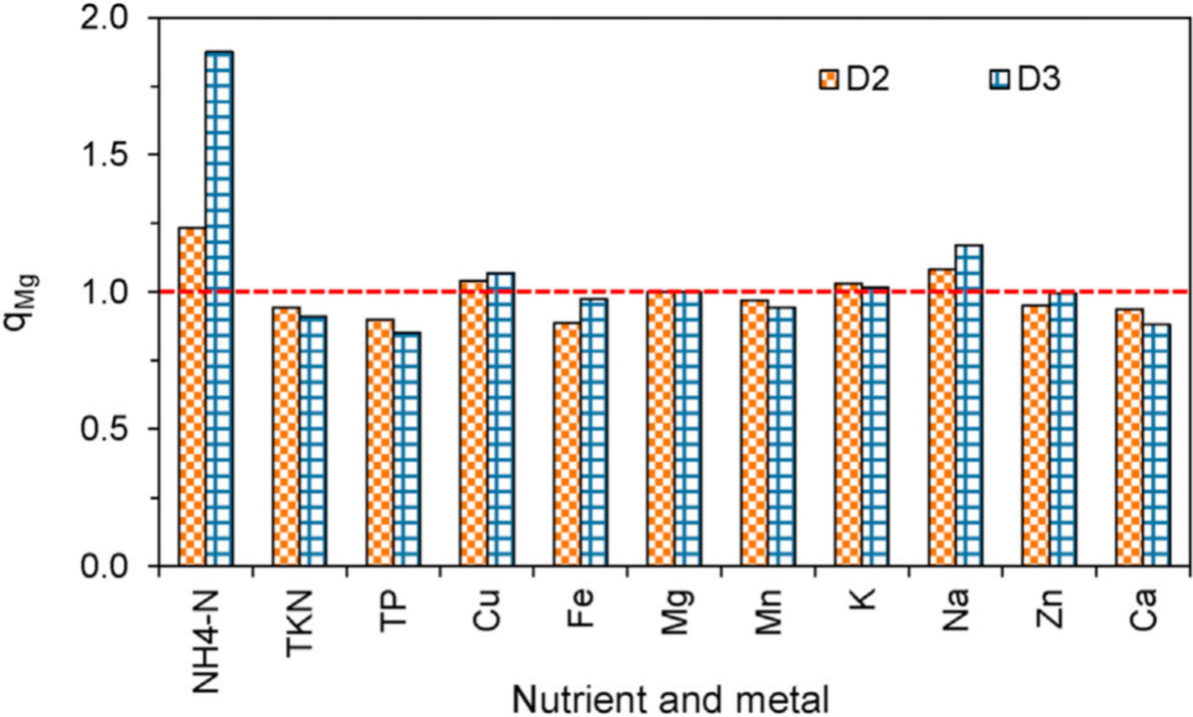
Accumulation or loss ratio quotient *q*_*Mg*_ of effluent nutrients and metals for D2 and D3.

Analysis with *q*_*Mg*_ in D2 and D3 nutrients also demonstrated an accumulation in NH_4_-N during AD. The *q*_*Mg*_ for NH_4_-N in the effluent from D2 and D3 was 1.23 and 1.88, respectively (Fig. 1). Organic matter was decomposed, and organic N could be released in inorganic form during AD. This could increase effluent NH_4_-N concentrations. The higher *q*_*Mg*_ for NH_4_-N in D3 effluent indicated a higher degradation of the organic matter in D3, probably due to the longer HRT of 34 days.

The *q*_*Mg*_ for TKN in the effluent from D2 and D3 were 0.94 and 0.91, respectively, and were lower than 1.0, indicating the loss in TKN concentrations during AD. These results agreed with the findings by Möller and Müller (2012). Part of the TKN could be converted to NH_4_-N because TKN is the sum of NH_3_, NH_4_-N, and organic nitrogen. It was reported that N loss during the AD process was most like due to loss of NH_3_ (Zirkler et al., 2014). Ammonia is a minor component of biogas and can reach several hundreds ppm in biogas (Strik et al., 2006). However, most research so far have focused on NH_3_ inhibition in anaerobic fermentation (e.g., Nielsen and Angelidaki, 2008), and little has been done on N loss via biogas inside digesters.

The *q*_*Mg*_ for TP in the effluent from D2 and D3 was 0.90 and 0.85, respectively, demonstrating relatively more loss in TP from digester influent to effluent compared with TKN. During the AD processes, an increase in pH could cause precipitation of P as Ca_3_(PO_4_)_2_ (Haygarth and Sharpley, 2000). This could explain the decrease in effluent TP in D3, in which the average pH was 6.1 ± 0.4 in influent and 7.9 ± 0.4 in the effluent (Table 2), but could not explain the same in D2, in which the average pH was 7.9 ± 0.3 in influent and 7.6 ± 0.2 in the effluent. The decrease in effluent TP in D2 could possibly be related to precipitations of struvite (NH_4_MgPO_4_∙6H_2_O) (Banks et al., 2011) and adsorption on solid surfaces in certain digesters (Marti et al., 2008).

The method of ratio quotient *q*_*Mg*_ was found suitable for D2 and D3. The changes in the Mg concentrations between the digester influent and effluent were 3.7% for D2 and 1.3% for D3. Magnesium remained as a relatively stable element during AD for the two digesters. In comparison, this difference in Mg concentrations for D1 was 20%, much larger than those for D2 and D3. This might be related to the different operation of D1 compared with the other two digesters. Thus, *q*_*Mg*_ was only used in D2 and D3 data analysis.

#### 3.3.4. Changes in metals

Anaerobic digestion brought changes in metal concentrations as well for the three digesters. Compared with respective influent (Table 3) and effluent (Table 5), the concentrations of eight metals in D1 increased from 17.1% (Ca) to 21.7% (K), except for Cu, which was decreased by 6.7%. Contrarily, most of the metal concentrations decreased in D2 and D3. Except for Na, which increased by 5.8%, all metals in D2 decrease from 3.7% (Mg) to 15.0% (Fe) in effluent. However, because of large standard deviations in the 2-year samples, all these changes were not statistically significant (*p* > 0.05).

The results of *q*_*Mg*_ for effluent metals from D2 and D3 showed losses in Fe, Mn, Zn, and Ca, but accumulations in Cu, K, and Na during the AD (Fig. 1). However, compared with nutrients, the accumulations and losses in metals were smaller and underwent relatively minor changes during AD.

There is little literature information available for the metal turnover in dairy manure and co-digestion digesters. The significant losses in Mn and Zn have also been observed by Massé et al. (2007), who reported that on average, 21% of Mn and 18% of Zn were retained in digesters. Because of the complexity of the AD process, the accumulations or losses in Cu, Fe, and K were different between the two digesters. The metals might be involved in many physicochemical processes (e.g., the precipitation of metals by S^2−^, ${\text{CO}}_{3}^{2-}$, and ${\text{PO}}_{4}^{3-}$), complexation reactions, and sorption to the solid fractions (Callander and Barford,1983a, b). Metals, such as Fe, might form precipitates as Fe(OH)_2_ or FeCO_3_, which might relate to the increased pH and decreased solubility of metals in anaerobic digesters. The conditions and the complicated reactions that occurred during AD can cause a variety of nutrient and metal characteristics of digester effluent. To avoid high applications of metals on a small area of land, appropriate digester effluent management is needed (Möller and Müller, 2012).

### 3.4. Relationship among effluent constituents

Effluent nutrients and metals showed close correlations with the effluent TS. All simple linear regression models based on TS to demonstrate nutrient and metal concentrations had coefficient of determination (R^2^) larger than 0.58 (Table 6). During the AD, a reduction in TS of the influent occurred because of the biodegradation of organic matter (Güngör and Karthikeyan, 2005. Thus, the stability of nutrients and metals in solid phase could also be enhanced. For example, most of the digester effluent TP was insoluble and allocated to the solid phase; but 45e80% of the N was in the liquid phase and presented as NH_4_-N (Möller and Müller, 2012). Therefore, it was reasonable to use TS to demonstrate digester effluent nutrient and metal concentrations.

However, when taking both the effluent TS and pH into account and using the multiple-linear regression models, the R^2^ of the models was significantly improved (*p* < 0.001) for TKN, TP, and NH_4_-N compared with only using TS (Table 6). The best-fit correlation R^2^ was 0.72 for TKN, 0.71 for TP, and 0.64 for NH_4_-N. The best fit multiple correlations for K, Na, and Fe with TS and pH yielded R^2^ of 0.66, 0.60, and 0.69, respectively (Table 6). The results demonstrated that multiple linear regressions were better than simple linear regressions for nutrient and metal concentration estimation. Similar conclusions were made in estimating nutrients in chicken manure using single and multiple linear equations based on dry matter and/or pH (Huang et al., 2011). The improved regressions with both TS and pH were because pH was expected to be a key factor affecting the chemical speciation of nutrients and metals during AD (Callander and Barford, 1983a).

Some of multiple linear regressions in Table 6 show a potential of using easily determinable parameters TS and pH to estimate NH_4_-N, TKN, TP, K, Na, and Fe concentrations in digester effluent. This could supplement laboratory analysis of nutrients and metals in the effluent. However, considering the complexity and variety of nutrient and metal characteristics of digester effluent, further investigation is needed to develop better models with higher accuracy and for different digesters.

## 4. Conclusions

Long-term performances to convert agricultural and agro-industrial wastes into organic fertilizer varied among the three digesters. They were mainly affected by types of feedstocks (dairy manure only vs. co-digestion wastes) and HRT (from 9 to 34 days) of digestion. Mono-feedstock D1 and D2 demonstrated similar changes in pH from influent to effluent. These changes were significantly different from co-digestion D3, although all digesters’ effluent pH was within the pH range in normally functioning anaerobic digesters. Compared with the influent, the reduced effluent pH for D1 (−0.6) and D2 (−0.3) and the raised effluent pH (+1.8) for D3 could potentially reduce or increase, respectively, N loss via NH_3_ emission during post-digestion storage and cropland application. Whereas D1 digestion did not result in changes in TS (*p* > 0.05), digestion in D2 and D3 significantly removed TS from influent by 26.5% (*p* < 0.05) and 39.0% (*p* < 0.01), respectively. The higher removal rates could be related to longer digester HRT. All three digesters significantly increased NH_4_-N concentrations, ranging from 21.4% to 81.8% (*p* < 0.05), but insignificantly changed TKN and TP concentrations (*p* > 0.05). The largest increase in NH_4_-N was from the D3 of the longest HRT. Most of the eight metals increased in D1, but decreased in D2 and D3 during the AD process. However, all the metal concentration changes were statistically insignificant (*p* > 0.05). The use of CRTS and *q*_*Mg*_ provided alternative approaches to obtain insights into AD conversion of nutrients and metals. Nutrients and three metals (K, Na, and Fe) demonstrated regression relationships with pH and TS (R^2^ ≥.60).

## Figures and Tables

**Table 1:** Basic information of the three anaerobic digesters in Midwest, USA.

Digester	D1	D2	D3
Digester design	Plug flow	Mixed Plug-Flow	Mixed Plug-Flow
Volume (m^3^)	5700	28,400	27,300
Hydraulic retention time (d)	9	12	34
Digestion temperature (°C)	35–37	38	38
Manure influent source	Dairy	Dairy	Mixed
Co-digestion influent source	No	No	Agro-industrial wastes

**Table 2:** Mean ± standard deviation of pH and total solids (TS) of the influent to and effluent from the three digesters.

	D1	D2	D3
Sampling event (n)	10	12	12
Influent pH	7.9 ± 0.3^A^	7.9 ± 0.3^A^	6.1 ± 0.4^B^
Effluent pH	7.3 ± 0.5^A^	7.6 ± 0.2^A^	7.9 ± 0.4^B^
Inf. vs eff. pH (*p*)	<0.01	<0.01	<0.01
Influent TS (%)	3.4 ± 1.0^A^	4.9 ± 1.1^B^	12.3 ± 3.2^C^
Effluent TS (%)	3.5 ± 1.0^A^	3.6 ± 1.3^A^	7.5 ± 1.7^B^
Inf. vs eff. TS (*p*)	>0.05	<0.05	<0.01

A, B, C: different superscript letters mean statistically different (*p* < 0.05) for values in the same row.

**Table 3:** Mean ± standard deviation of nutrient and metal concentrations and CRTS in the influent to the three digesters.

Nutrient or metal	Concentration (mg L^−1^)			CRTS (mg g^−1^)		
		
	D1	D2	D3	D1	D2	D3
NH_4_-N	641 ± 109^a^	1035 ± 184^b^	2125 ± 520^c^	20.0 ± 5.8^A^	22.0 ± 3.7^A^	18.6 ± 6.9^A^
TKN	1743 ± 414^a^	2495 ± 555^b^	8595 ± 3337^c^	52.8 ± 11.6^A^	52.4 ± 8.4^A^	74.9 ± 40.1^A^
TP	298 ± 66^a^	417 ± 105^b^	2933 ± 740^c^	9.1 ± 2.0^A^	8.7 ± 1.5^A^	24.8 ± 7.2^B^
Cu	18.8 ± 9.0^a^	14.2 ± 4.0^a^	4.7 ± 0.9^b^	0.5 ± 0.2^B^	0.3 ± 0.1^A^	0.04 ± 0.01^C^
Fe	44.5 ± 10.6^a^	60.6 ± 15.6^b^	1462 ± 397^c^	1.4 ± 0.3^A^	1.2 ± 0.2^A^	12.4 ± 4.1^B^
Mg	485 ± 89^a^	651 ± 125^b^	544 ± 175^a,b^	14.7 ± 2.3^A^	13.6 ± 1.9^A^	4.6 ± 1.4^B^
Mn	12.7 ± 2.3^a^	16.3 ± 4.1^b^	13.1 ± 2.4^a^	0.4 ± 0.1^A^	0.3 ± 0.0^A^	0.1 ± 0.0^B^
K	1157 ± 202^a^	1485 ± 315^b^	4243 ± 3791^c^	35.5 ± 7.2^A^	31.4 ± 6.4^A^	30.9 ± 17.6^A^
Na	549 ± 94^a^	614 ± 111^a^	1984 ± 628^b^	16.7 ± 2.5^B^	13.0 ± 2.2^A^	17.1 ± 7.0 ^A,B^
Zn	14.4 ± 4.0^a^	26.3 ± 8.5^b^	27.0 ± 6.6^b^	0.4 ± 0.2^A^	0.5 ± 0.1^A^	0.2 ± 0.1^B^
Ca	1024 ± 196^a^	1270 ± 269^b^	1864 ± 717^c^	31.1 ± 5.8^A^	26.4 ± 3.4^A^	16.4 ± 8.6^B^

Sample event n: D1 = 10, D2 = 12, and D3 = 12.

“a”, “b”, and “c” Different superscripts mean statistically different (*p* < 0.05) for concentrations in the same row.

“A”, “B”, and “C” Different superscripts mean statistically different (*p* < 0.05) for CRTS (concentration related to total solids) in the same row.

**Table 4:** Manure digester effluent nutrient and metal concentrations reported in the literature.

Feedstock	pH	NH_4_-N (mg L^−1^)	TKN (mg L^−1^)	TP (mg L^−1^)	Cu (mg L^−1^)	Fe (mg L^−1^)	Mg (mg L^−1^)	Mn (mg L^−1^)	Zn (mg L^−1^)	Ca (mg L^−1^)
Chicken litter ^a^		3335	3338	158						
Chicken litter ^b^	8.0–8.5	1600–3200		50–300						
Cattle manure ^c^	8.2	2119	3254	715						
Cattle manure ^d^	7.7	840	1123	128						
Cattle manure ^e^	8.4	1328–2892	1730–3450	51–213						
Cattle manure ^f^	6.9, 8.0	453			0.1				0.5	
Cattle manure ^g^		123		338			153			
Pig manure ^h^	8.1	1500	2700	800						
Pig manure ^i^	8.4	3423	4276	526	7.0				56.0	
Pig manure ^j^	7.1		323	88		29.3	12	21.7		81
Pig manure ^k^	7.1	1171		153	1.0	2.7	33	1.2	1.1	109
Pig manure ^l^	8.37	1672		186						
Pig manure ^m^	8.23	780			2.7				9.9	
Cattle manure + GL^n^	6.5	770	1600	330	8.4	126.0	226	11.3	19.0	1165
Cattle manure + AR^n^	7.8	1370	2300	400	5.6	123.0	423	13.5	14.5	2023
Pig manure + CR ^n^	7.9	2733	3500	1133	7.7	174.0	684	25.8	52.5	1942
Pig manure + AB^n^	8.0	3033	3933	500	11.1	45.0	252	9.9	86.4	615
Mean	7.8	1666	2635	420	5.5	83	255	13.9	30.0	989
Standard deviation	0.6	1094	1283	311	3.9	67	235	8.8	31.2	866

GL: glycerine. AR: agro-industrial residue. CR: crop residue. AB: animal byproducts.

a Huo (2011).

b Wang et al. (2012b).

c Martin (2005).

d Martin (2008).

e Zheng et al. (2011).

f Ke et al. (2014).

g Castro-Molano et al. (2018).

h Loria and Sawyer (2005).

i Martin (2003).

j Tang et al. (2010).

k Wang et al. (2012a).

l Zheng et al. (2019).

m Tang et al. (2020).

n Alburquerque et al. (2012).

**Table 5:** Mean ± standard deviation of effluent nutrient and metal concentrations and CRTS from the three digesters.

Nutrient or metal	Concentration (mg L^−1^)			CRTS (mg g^−1^)		
		
	D1	D2	D3	D1	D2	D3
NH_4_-N	877 ± 182^a^	1257 ± 295^b^	3863 ± 888^c^	27.4 ± 10.0^A^	37.4 ± 9.1^B^	53.1 ± 12.5^C^
TKN	1925 ± 391^a^	2315 ± 567^a^	7440 ± 1434^b^	57.5 ± 12.1^A^	67.6 ± 15.6^A^	102.2 ± 21^B^
TP	313 ± 60.3^a^	384 ± 124^a^	2500 ± 510^b^	9.4 ± 2.2^A^	11.0 ± 3.3^A^	34.3 ± 7.3^B^
Cu	17.6 ± 7.0^a^	13.5 ± 2.4^a^	4.8 ± 1.3^b^	0.5 ± 0.1^A^	0.4 ± 0.1^A^	0.1 ± 0.0^B^
Fe	52.4 ± 12.1^a^	51.5 ± 12.8^a^	1270 ± 286^b^	1.6 ± 0.4^A^	1.5 ± 0.3^A^	17.5 ± 4.2^B^
Mg	581 ± 126^a^	627 ± 104^b^	551 ± 213^c^	17.2 ± 3.2^A^	18.5 ± 3.4^A^	7.6 ± 3.2^B^
Mn	14.6 ± 2.9^a^	15.6 ± 4.4^b^	12.3 ± 3.2^c^	0.4 ± 0.1^A^	0.4 ± 0.1^A^	0.2 ± 0.1^B^
K	1408 ± 311^a^	1475 ± 259^a^	3432 ± 1554^b^	42.0 ± 9.0^A^	43.9 ± 9.8^A^	45.6 ± 16.9^A^
Na	652 ± 158^a^	649 ± 116^a^	2058 ± 553^b^	19.3 ± 3.8^A^	19.4 ± 4.8^A^	28.3 ± 8.2^B^
Zn	17.1 ± 5.0^a^	25.2 ± 9.1^b^	26.5 ± 7.0^b^	0.5 ± 0.1^A^	0.7 ± 0.2^B^	0.4 ± 0.1^A^
Ca	1206 ± 230^a,b^	1155 ± 249^a^	1489 ± 456^b^	36.1 ± 7.8^A^	33.6 ± 6.2^A^	20.7 ± 7.3^B^

Sample event n: D1 = 10, D2 = 12, and D3 = 12.

“a”, “b”, and “c” Different superscripts mean statistically different (*p* < 0.05) for concentrations in the same row.

“A”, “B”, and “C” Different superscripts mean statistically different (*p* < 0.05) for CRTS (concentration related to total solids) in the same row.

**Table 6:** Regression equations for the nutrients and selected metals in the digester effluent.

Nutrient & metal (mg L^−1^)	Independent variable	Regression equation	R^2^
NH_4_-N	TS	NH_4_-N = 478TS − 294	0.58
	pH, TS	NH_4_-N = 849 pH + 421TS − 6460	0.64
TKN	TS	TKN = 952TS − 690	0.67
	pH, TS	TKN = 1553 pH + 847TS − 11966	0.72
TP	TS	TP = 374TS − 738	0.65
	pH, TS	TP = 656 pH + 330TS − 5502	0.71
K	TS	K = 459TS − 120	0.63
	pH, TS	K = 612 pH + 418TS − 4565	0.66
Na	TS	Na = 249TS − 83	0.58
	pH, TS	Na = 277 pH + 230TS − 2097	0.60
Fe	TS	Fe = 208TS − 544	0.63
	pH, TS	Fe = 385 pH + 182TS − 3337	0.69

TP: total phosphorus. TS: total solids (%).
